# ERK2 and Akt are negative regulators of insulin and Tumor Necrosis Factor-α stimulated VCAM-1 expression in rat aorta endothelial cells

**DOI:** 10.1186/s12950-016-0115-6

**Published:** 2016-02-25

**Authors:** Gregory B. Pott, Mark Tsurudome, Nadia Bamfo, Marc L. Goalstone

**Affiliations:** Division of Endocrinology, Metabolism and Diabetes, University of Colorado Anschutz Medical Campus, 12801 East 17th Avenue. Mail Stop: 8106, Aurora, CO 80045 USA; Eastern Colorado Health Care System, (Denver VA Medical Center), 1055 Clermont Street. Mail Stop 151, Denver, CO 80220 USA

**Keywords:** Atherosclerosis, Inflammation, ERK2, Akt, RNAi, Insulin, TNFα, VCAM-1

## Abstract

**Background:**

Diabetes is quickly becoming the most widespread disorder in the Western world. Among the most prevalent effects of diabetes is atherosclerosis, which in turn is driven in part by inflammation. Both insulin and Tumor Necrosis Factor-alpha (TNFα) increase the presence of Vascular Cellular Adhesion Molecule-1 (VCAM-1) expression. The aim of this study is to determine the effects of downregulating Extracellular signal-Regulated Kinase-2 (ERK2) and Akt on insulin and TNFa-stimulated VCAM-1 expression.

**Methods:**

Here we begin to define the relationships between ERK2 and Akt regulation of insulin and TNFα-stimulated VCAM-1 expression in Rat Arterial Endothelial Cells (RAEC) by transfecting RAEC with ERK2 and Akt RNA interference (RNAi) and then treating these cells with insulin (10 nM) or TNFα (10 ng/mL) alone or in combination.

**Results:**

Western blot analyses, flow cytometry and confocal microscopy were used to determine changes in VCAM-1 expression within the above-stated parameters. Cells transfected with ERK2 or Akt RNAi plasmids increased insulin and TNFα-stimulated VCAM-1 total protein expression significantly (*P* < 0.05) greater than that seen in mock transfected cells and expressed cell surface VCAM-1 greater than that seen in mock transfected cells as indicated by flow cytometry and confocal microscopy. Nevertheless, the decrease of both kinases did not increase insulin or TNFα-stimulated VCAM-1 expression above that seen when one or the other RNAi was present.

**Conclusions:**

Taken together, our results demonstrate that ERK2 and Akt *may* be negative regulators of insulin and TNF-α stimulated VCAM-1 and that their loss or down regulation might upregulate VCAM-1 expression and contribute to vascular disease.

## Background

Diabetes mellitus (DM) is a serious global health problem. In 2014, 29.1 million adults were clinically diagnosed with diabetes and $245 billion dollars were spent on the treatment for diabetes in the United States (US) alone. Diabetes brings along with it a sequela of adverse conditions such as, but not limited to, insulin resistance, dyslipidemia, retinopathy, nephropathy, and cardiovascular disease (CVD) [1]. CVD is among the most aggressive aspects of DM and is associated with plaque formation, decreased blood flow rate, thrombosis, occlusion and rupture of the arteries, morbidity and mortality [2].

Atherosclerosis has been linked to a range of cellular and molecular changes in the vascular wall most of which are noted in the endothelial cells of the arteries [3]. Associated with the remodeling of the arterial walls is the expression of the endothelial surface molecule, Vascular Cell Adhesion Molecule-1 (VCAM-1) [4]. VCAM-1 appears to be upregulated in DM *patients’ endothelial cells*. The *increase of* VCAM-1 appears to foster the recruitment of monocytes to the surface of the endothelium and their subsequent transmigration across the endothelial layer [5]. Once embedded into the vascular smooth muscle layer, these monocytes mature to fully functional macrophages, which subsequently activate and express inflammatory cytokines such as Tumor Necrosis Factor-alpha (TNFα) [4].

Hyperinsulinemia (HI) and increased presence of TNFα are consequences of insulin resistance [1, 6]. Both HI and TNFα in turn exacerbate the pathophysiological conditions of the endothelium and cause an increase in VCAM-1 expression. Expression of VCAM-1 is regulated by a family of kinases, which mediate external signals to internal events [7, 8]. These kinases include, but are not limited to, extracellular signal-regulated kinase 1/2 (ERK1/2), protein kinase B/Akt (Akt), p38 kinase and c-Jun N-terminal kinase (JNK). ERK2 and Akt are major mediators of external signals to internal events. *Others have shown that Akt and ERK2 contribute to the regulation of VCAM-1* [9, 10]. *Thus, we started our studies examining these two kinases and their effects on VCAM-1 expression.* Here we report that the reduction of ERK2 or Akt in rat aorta endothelial cells (RAEC) increases insulin and TNFα-stimulated total VCAM-1 expression. In contrast, the simultaneous reduction of both ERK2 and Akt did not cause an additive *increase in* VCAM-1 protein above that of ERK2 or Akt alone. Although there may not be a cumulative effect of increased VCAM-1 due to the decreased presence of ERK2 and Akt in endothelial cells, their diminished presence may play a significant role in the inflammatory attributes of cardiovascular disease.

Here we report that when the expression of ERK2 and Akt was decreased via RNA interference, we observed insulin and TNFα-stimulated *increased* VCAM-1 expression at the protein and cell surface level. However, simultaneous downregulation of both ERK2 and Akt did not show an additive or synergistic effect.

## Methods

### Materials

All general lab reagents were purchased from Sigma-Aldrich (St. Louis, MO). PVDF membranes and other Western blot accessories were from GE Healthcare/Amersham (Piscataway, NJ). Primary rabbit antibodies to ERK1/2 (9102), Akt (4056), and alpha-tubulin (2144S) were from Cell Signaling (Boston, MA). The primary rabbit antibody to VCAM-1 (NBP1-95622) was from Novus Biologicals (Littleton, CO) and goat anti-rabbit-secondary antibody IRDye680RD (926–68171) was from LI-COR (Lincoln, NE). Rat aorta vascular endothelial cells (RAEC) (CRL-1444) were from ATCC (Manassas, VA) and culture medium was from Life Technologies (Grand Island, NY). ERK2 (KR48780P) and Akt (KR45425P) *short hairpin (sh)* RNA plasmids were obtained from SA Biosciences/Qiagen (Valencia, CA). Transfection Medium (108062) and Reagent ((108061) were from Santa Cruz Biotechnology (Dallas, Tx). DyLight 488-conjugated anti-VCAM-1 antibody was from Thermo Scientific (Pittsburgh, PA). Four-well chamber slides were from Thermo Fisher and DAPI Mounting Medium was from Vector Labs (Burlingame, CA). *Insulin (19278) was from Sigma-Aldrich (St. Louis, MO) and TNFα (11271156001) was from Roche (Indianapolis, IN).*

### Cell culturing

RAEC were cultured in complete growth medium (CGM) [DMEM with 4 mM L-glutamine, 4.5 g/L-glucose and 1.5 g/L sodium bicarbonate) and supplemented with 10 % heat-inactivated fetal bovine serum (HI-FBS) (10438–026) (Life Technologies, Grand Island, NY) and 1 % Antimycotic-Antibiotic solution (15240–062) (Life Technologies) and cultured at 37 °C, 5 % CO_2_ atmosphere.

### Preparation of shRNA stable cell lines

RAEC were grown to 50–70 % confluence in CGM in 6-well culture plates. Cells were transfected with shERK2 *(clone #2)* or shAkt *(clone #2)* inhibitory plasmids as previously described [11]. Cells were incubated in CGM containing 2 μg/mL of Puromycin (Sigma-Aldrich) for 2–3 weeks for selection of Puromycin resistant transformants.

### Dual transfection of stable cell lines

To examine the effect of simultaneous ERK2 and Akt knockdown on VCAM-1 expression, the ERK2 shRNA stable cell line (ERK2 KD) was transiently transfected with shAkt plasmid and the Akt shRNA stable cell line (Akt KD) was transiently transfected with the shERK2 plasmid. These two protocols were carried out in order to see if any difference occurred with respect to transfection sequence. Stable cell lines were transiently transfected with shRNA plasmid DNA as described above and incubated for 5 h with the DNA transfection mix. Subsequently the transfection mix was aspirated and replaced with 2.0 mL CGM. Stimulation of cells by insulin and/or TNFα occurred 48 h after transient transfection was accomplished.

### Stimulation of VCAM-1 expression

RAEC were cultured in CGM, whereas shRNA stable cell lines (e.g., ERK2 KD and Akt KD) were cultured in CGM containing 2 μg/mL Puromycin until assays were performed. After incubating the transfected cells for an additional 48 h, the cells were stimulated with or without insulin (10 nM) and in the presence or absence of TNFα (10 ng/mL) and evaluated for VCAM-1 expression as previously described [11]. Briefly, TNFα stimulation occurred over a total of 6 h and cells activated with insulin were stimulated for 1 h.

### Western blot analysis

Sodium Dodecyl Sulfate Polyacrylamide Electrophoresis was performed on cleared lysates. Western blot analysis was subsequently performed as previously described, [11] with the following differences. After completion of protein transfer, membranes were washed in ultra-pure water for 5 min. Membranes were then incubated in 3 % non-fat milk (milk) in Tris-buffered Saline (TBS) blocking solution for 1 h at room temperature and then incubated with a designated primary antibody solution (1:1000 in 3 % milk/TBS-T) overnight at 4 °C. Membranes were washed 4 times with TBS plus Tween (TBS-T) for 5 min at room temperature and then incubated with a goat anti-rabbit secondary antibody (1:5000 in 3 % milk/TBS-T) conjugated to fluorochrome IR680RD for 1 h at room temperature. Membranes were washed 4 times with TBS-T for 5 min at room temperature and then incubated with a rabbit anti-tubulin primary antibody solution (1:1000 in 3 % milk/TBS-T) for 3 h at room temperature. After washing the membranes for 4 times with TBS-T, the membranes were again incubated with a goat anti-rabbit secondary antibody (1:5000 in 3 % milk/TBS-T) conjugated to fluorochrome IR680RD for 1 h at room temperature. The membranes were washed 4 times with TBS-T and allowed to dry before performing densitometry. Densitometry was performed using an Odyssey Licor system (Lincoln, NE). Alpha-tubulin protein was used to normalize VCAM-1 signals.

### Flow cytometry

Non-transfected RAEC were used as controls. Stably transfected RAEC Akt knockdown (Akt KD) and ERK2 knockdown (ERK2 KD) cell lines were inoculated into 6-well tissue culture dishes, transiently transfected with shERK2 or shAkt plasmids, respectively, and stimulated with insulin, TNFα, or combined insulin and TNFα as described above in Dual Transfection. The cells were washed twice with 2 mL of 1X PBS (Gibco). The PBS was aspirated and 0.5 mL of Cell Dissociation Solution Non-Enzymatic (Sigma-Aldrich) was added to each well. After incubating the cells at 37 °C and 5 % CO_2_ for 30 min, 1 mL of 1 % Bovine Serum Albumin (BSA, Sigma-Aldrich) in PBS was added to the cells and then were gently triturated into a single cell suspension. The cells were transferred to 5 mL Falcon polystyrene round bottom tubes (Thermo Scientific) and centrifuged at 500 x g for 5 min. After aspirating the supernatants, the cells were resuspended in 3 mL 1 % BSA, pelleted at 500 × g by centrifugation, and the supernatants removed by aspiration. The cells were resuspended in 200 μL of 1 % BSA. Two microliters of DyLight 488-conjugated anti-VCAM-1 antibody (Life Technologies, Grand Island New York) were added to each tube and the cells were resuspended by vortexing. The cells were incubated in the dark for 30 min at room temperature. The cells were centrifuged, washed twice with 3 mL 1 % BSA and resuspended in 200 μL of 1 % paraformaldehyde (PFA, Electron Microscopy Sciences, Hatfield, PA). After incubating the cells for 5 min at room temperature, the cells were diluted with an additional 300 μL of PBS and analyzed using flow cytometry.

The experiments were run on a BD LSRII (BD Biosciences, San Jose, CA). MFI and gating percentages as part of data analysis were performed using BD FACSDiva v6 software.

### Chamberslide cell preparation

2 × 10^5^ of control RAEC, Akt KD or ERK2 KD stable cell lines were plated in 1 mL of CGM in each well of a 4-well chamberslide and allowed to grow for 24 h, 37 °C and 5 % CO_2_. The medium was aspirated and 1.0 mL of fresh CGM was applied to the cells. Akt KD and ERK2 KD were either mock transfected, or transiently transfected with shERK2 or shAkt, respectively for 48 h. Cells were then treated with either vehicle, 10 ng/mL TNFα, insulin (10 nM) alone, or TNFα (10 ng/mL) and then insulin (10 nM). The medium was aspirated and the cells were washed three times with PBS and then incubated in 400 μL of 4 % paraformaldehyde in PBS for 30 min. The medium was aspirated and washed three times with 1 mL of PBS. The final PBS wash was aspirated and 400 μL of a 1:1000 DyLight anti-VCAM-1 antibody soluton in 1 % BSA was added to each chamber and incubated for 30 min at room temperature. The cells were then washed three times with 500 μL of 1 % BSA. The chamber walls were removed and one drop of DAPI Mounting Medium was added to each group of cells on the slide. Cells were then sealed with a glass cover slip using clear nail polish. Slides were kept in a dark refrigerator until microscopic visualization.

### Confocal microscopy

A single, non-confluent monolayer of cells were imaged with a Leica TSC SP8X white light laser scanning confocal microscope (Leica Microsystems GmbH. Ernst-Leitz-Straße 17–37 Wetzlar, 35578 Germany). Excitation of the DAPI channel was achieved using a 405 nm diode laser with an excitation intensity level of 2.67 %. Emission signal was captured with standard PMT Channel 1 with an emission gap of 430 nm - 480 nm. Excitation of ALexa fluor 488 utilized the Leica Supercontinuum white light laser visible excitation laser line (488 nm) and an intensity level of 3 %. Emission signal was captured with Leica HyD 2 detector (Hybird 2 PMT) with a with a set emission gap of (505 nm - 555 nm). All image acquisitions were carried out using the Leica Application Suite X (version 1.1.0.12420, LASX AF).

### Data analysis

Data were analyzed by either unpaired Student’s *t* test (two groups) or ANOVA with subsequent Tukey posttest (several groups) as indicated. A “P” value of less than 0.05 was considered significant. Results were expressed as the mean ± SEM of three or more independent experiments.

## Results

**Fig. 1:**
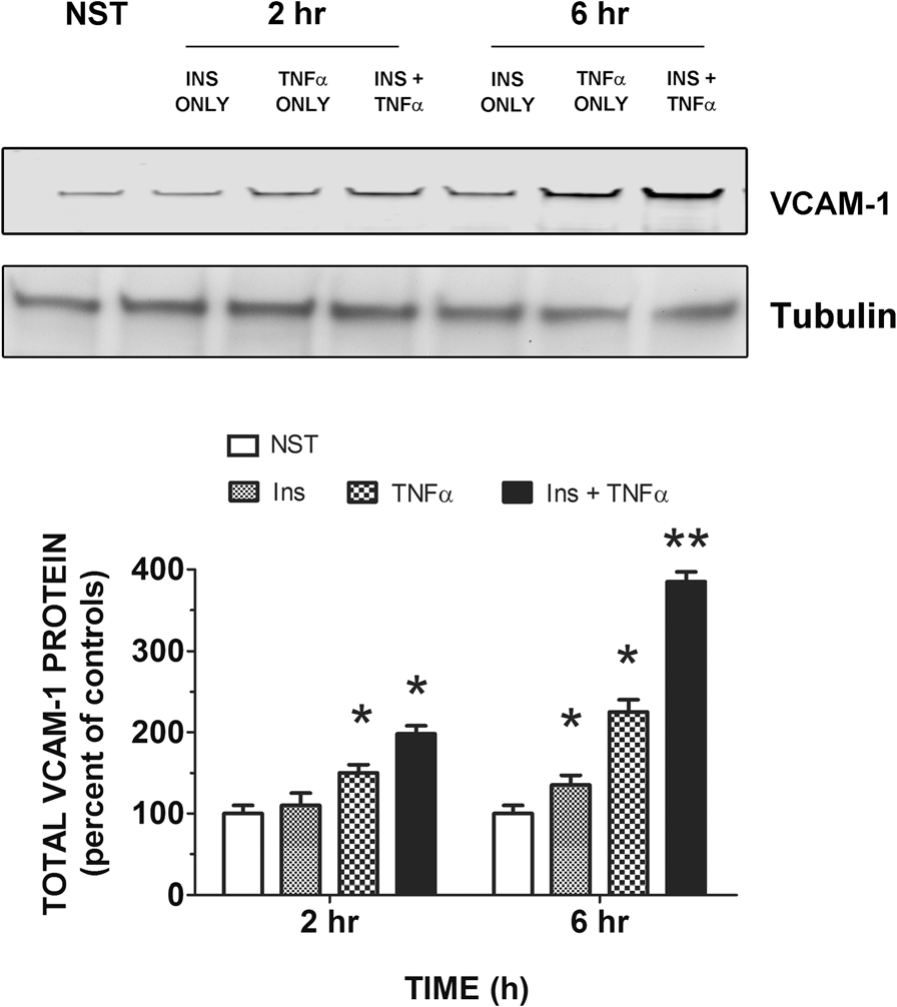
Insulin and TNFα increase expression of total VCAM-1 in rat aorta endothelial cells over time. RAEC were cultured in Complete Growth Medium (CGM) until 90 % confluent. Subsequently, cells were stimulated for indicated times with insulin (10 nM) or TNFα (10 ng/mL) alone or in combination. Total VCAM-1 protein was determined by Western blot analysis. Analysis was performed by the Licor Odyssey detection system. Total VCAM-1 is expressed as the percent of controls and represents the mean ± SEM of six independent experiments. *, *P* < 0.05 vs negative controls. **, *P* < 0.05 vs TNFα alone

Both insulin and TNFα significantly (*P* < 0.05) increased VCAM-1 at 6 h as compared to controls (Fig. 1). TNFα stimulated VCAM-1 was significantly (*P* < 0.05) greater than that seen for insulin alone at 2 and 6 h time points. Interestingly, cells stimulated with insulin plus TNFα for 6 h increased VCAM-1 significantly (*P* < 0.05) greater than that seen for insulin or TNFα alone.

In order to tease out the intracellular kinase regulators of these events, we incorporated RNA interference into these experiments to better understand which kinases were mediating the external signals of insulin and TNFα with regards to the expression of VCAM-1 protein.

In previous studies, we investigated the effect of ERK5 knockdown on insulin and TNFα-stimulated VCAM-1 [11]. In those studies, decreased ERK5 resulted in decreased insulin and TNFα-stimulated VCAM-1 expression. To continue this line of study we measured the expression of VCAM-1 in vascular cells in which expression of either ERK2 or Akt or both were decreased using RNAi techniques and in the presence of insulin and/or TNFα.

**Fig. 2:**
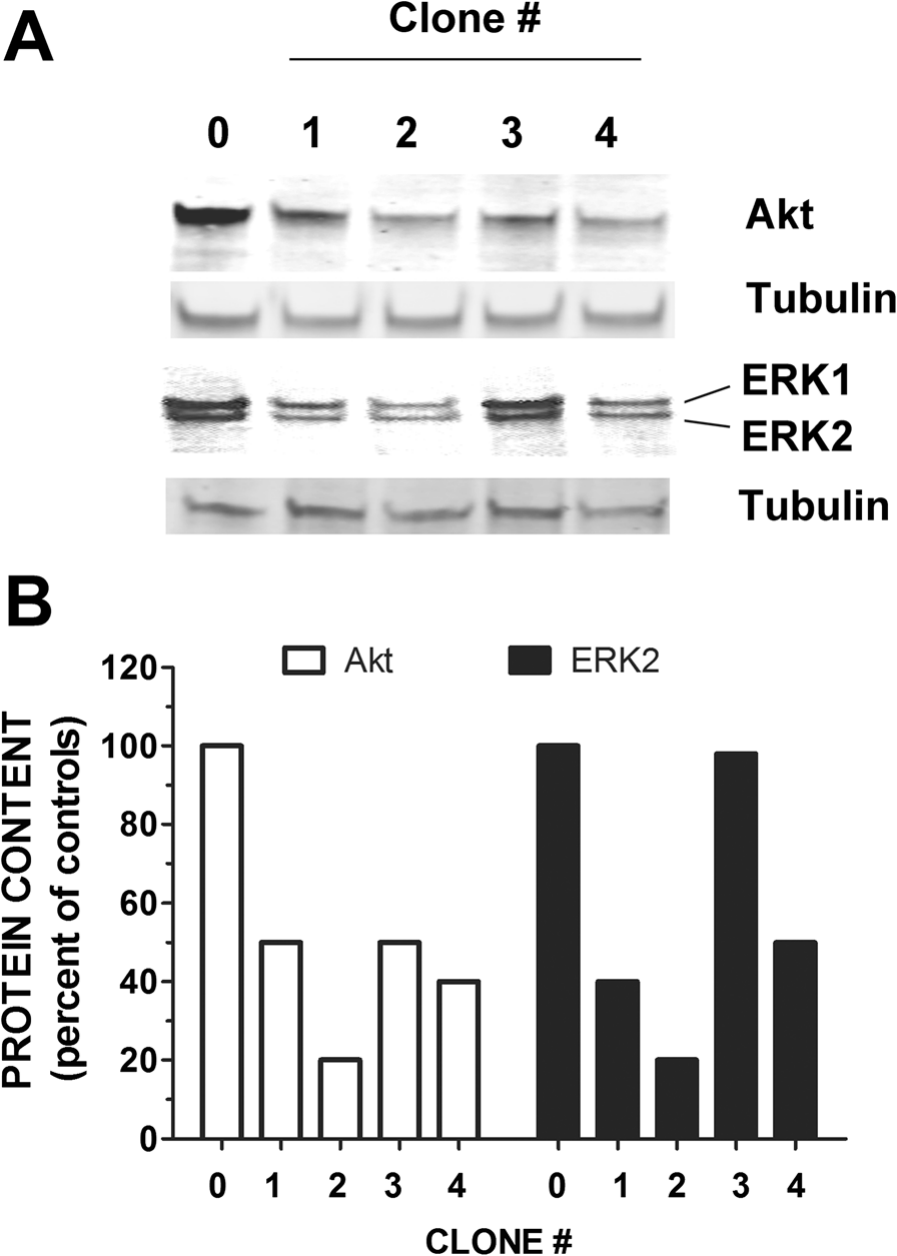
Different clones of shRNA for Akt and ERK2 differentially downregulate their respective proteins. Akt and ERK2 shRNA clones provided by manufacturer were amplified in E. coli and isolated by plasmid preparation. Individual clones of each shRNA were transfected into different groups of RAEC as described in Methods. (Panel **a**) Akt and ERK2 proteins were quantified in unstimulated cells by Western blot analysis in untreated cells. “0” is negative control clone, and numbers 1–4 are different clones provide by the manufacturer. Intensity of bands are proportional to expression of designated protein in the presence of each shRNA clone. (Panel **b**) Relative protein expression is compared to controls (negative control clone)

**Fig. 3:**
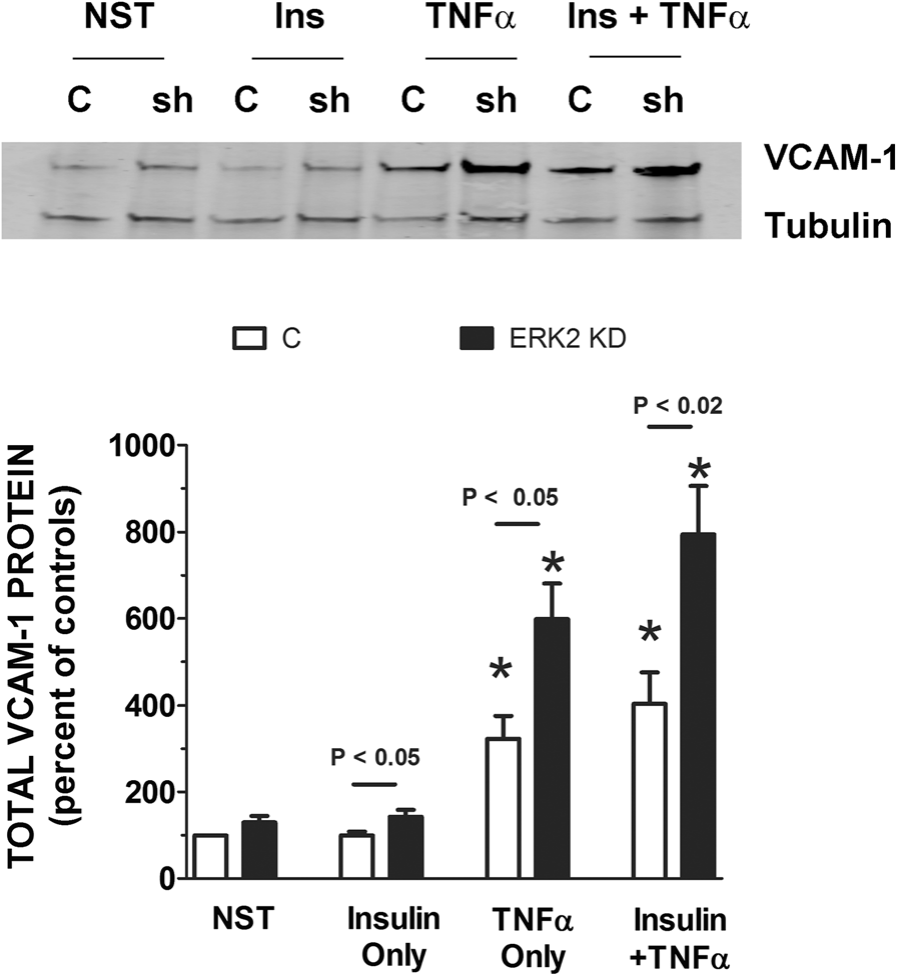
RAEC transfected with shERK2 *(clone #2)* and then stimulated with insulin or TNFα alone or in combination demonstrates the negative regulation of ERK2 in VCAM-1 expression. RAEC were stably transfected with shERK2 (ERK2 KD) and then stimulated with insulin (10 nM, 1 h) or TNFα (10 ng/mL, 6 h) alone or in combination. Total VCAM-1 protein was analyzed by SDS-PAGE and determined by Western blot analysis. (Upper Panel) “NST”, cells not stimulated with insulin or TNFα. “Ins” cells stimulated with insulin. “TNFα” cells stimulated with TNFα. “C” control cells not transfected with shRNA, while “sh” cells were transfected with shERK2. (Lower Panel) “C” control RAEC not transfected (open bars). ERK2 KD are RAEC stably transfected with shERK2 (black bars). “NST” cells not stimulated with insulin or TNFα. Total VCAM-1 is expressed as the percent of controls and represents the mean ± SEM of six independent experiments. Alpha-tubulin was used to normalize VCAM-1 profiles. *, *P* < 0.05 vs non-stimulated cells

After first determining which shERK2 and shAkt plasmid clone decreased its respective kinase most significantly (Fig. 2), we transfected the most efficacious shERK2 plasmid clone (*#2*) into our cells and treated with either insulin (10 nM) or TNFα (10 ng/mL) alone or in combination for designated times. Subsequently, we measured the change in total VCAM-1 protein between positive controls (no shRNA) and ERK2 KD cells. Interestingly, in the ERK2 KD cells, insulin-stimulated VCAM-1 increased 52 % above positive controls (cells treated with insulin with no shERK2) and TNFα-alone stimulated VCAM-1 84 % above positive controls (TNFα- stimulated cells with no shERK2) (Fig. 3). In ERK2 KD cells, VCAM-1 continued to increase 96 % above combined insulin plus TNFα-stimulated positive controls.

**Fig. 4:**
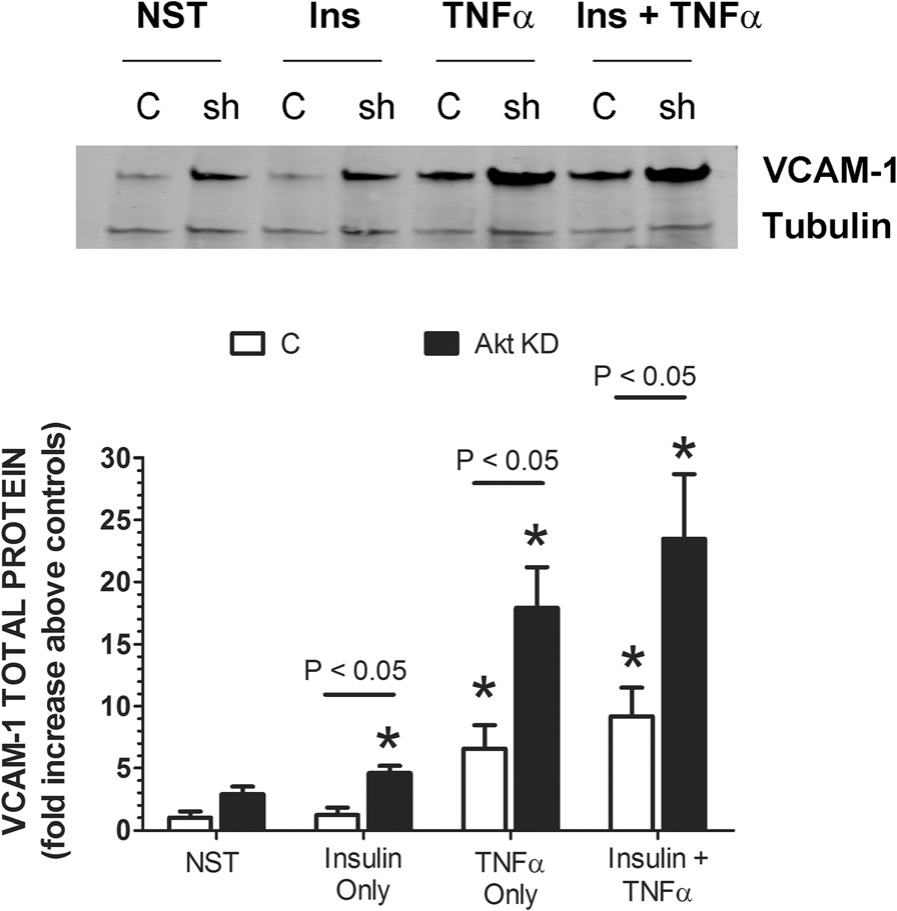
RAEC transfected with shAkt *(clone #2)* and then stimulated with insulin or TNFα alone or in combination demonstrates the negative regulation of Akt in VCAM-1 expression. REAC were stably transfected with shAkt (Akt KD) and then stimulated with insulin (10 nM, 1 h) or TNFα (10 ng/mL, 6 h) alone or in combination. Total VCAM-1 protein was analyzed by SDS-PAGE and determined by Western blot analysis. (Upper Panel) “NST”, cells not stimulated with insulin or TNFα. “Ins” cells stimulated with insulin. “TNFα” cells stimulated with TNFα. “C” control RAEC not transfected with shRNA, while “sh” cells were transfected with shAkt. (Lower Panel) “C” control RAEC not transfected (open bars). Akt KD are RAEC stably transfected with shAkt (black bars). “NST” cells not stimulated with insulin or TNFα. Total VCAM-1 is expressed as the percent of controls and represents the mean ± SEM of six independent experiments. Alpha-tubulin was used to normalize VCAM-1 profiles. *, *P* < 0.05 vs non-stimulated cells

We next determined whether shAkt affected insulin and TNFα-stimulated VCAM-1 expression. Similar to the results observed with ERK2 cells, the presence of shAkt (Akt KD) significantly increased VCAM-1 in the presence of insulin or TNFα alone 3.8-fold and 2.7-fold, respectively, above positive controls (cells not transfected with shRNA) (Fig. 4). In the presence of combined insulin and TNFα, Akt KD cells exhibited a 2.6-fold increase in VCAM-1 above positive controls cells treated with insulin and TNFα.

**Fig. 5:**
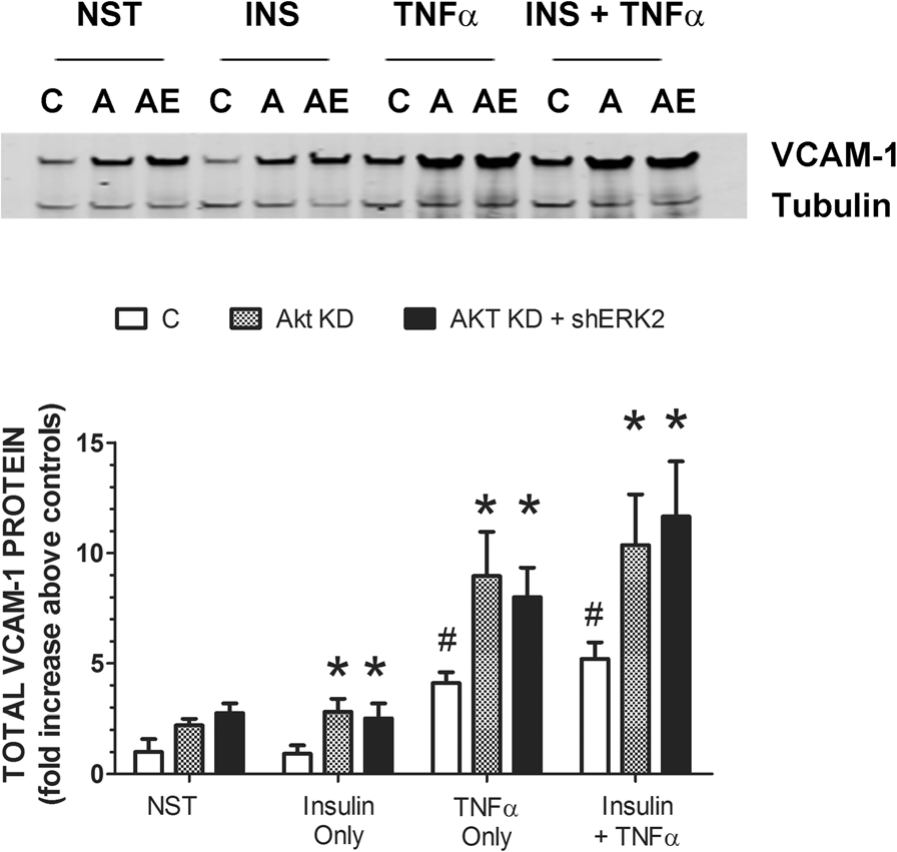
RAEC transfected with shAkt and shERK2 and then stimulated with insulin or TNFα alone or in combination exhibit increased VCAM-1 protein expression greater than stimulated, mock transfected cells, but not greater than cells transfected with shAkt alone. RAEC were stably transfected with shAKT and then transiently transfected with shERK2. Subsequently, cells were stimulated with insulin (10 nM, 1 h) or TNFα (10 ng/mL, 6 h) or in combination. (Upper Panel) C, control RAEC, no shRNA. A, cells transfected with shAkt alone. AE, cells stably transfected with shAkt and then transiently transfected with shERK2. (Lower Panel) Open bars are control cells not transfected. Stippled bars (Akt KD) RAEC stably transfected with shAkt. Black bars (Akt KD + shERK2) RAEC stably transfected with shAkt and transiently transfected with shERK2. “NST”, cells not stimulated with insulin or TNFα. Total VCAM-1 is expressed as fold increase above negative controls and represents the mean ± SEM of five independent experiments. Alpha-tubulin was used to normalize VCAM-1 profiles. #, *P* < 0.05 verses non-stimulated controls. *, *P* < 0.05 versus stimulated controls of similar treatment group without shRNA

Next, we were interested in changes in VCAM-1 expression in cells stimulated with insulin and/or TNFα and double transfected with shAkt and then shERK2. First, RAEC were stably transfected with shAkt (2 weeks before treatment) and then transiently transfected with shERK2 48 h before insulin and TNFα treatment. Interestingly, Akt KD cells transiently transfected with shERK2 exhibited no increases in insulin or TNFα stimulated VCAM-1 significantly greater than that seen in Akt KD cells alone (Fig. 5).

**Fig. 6:**
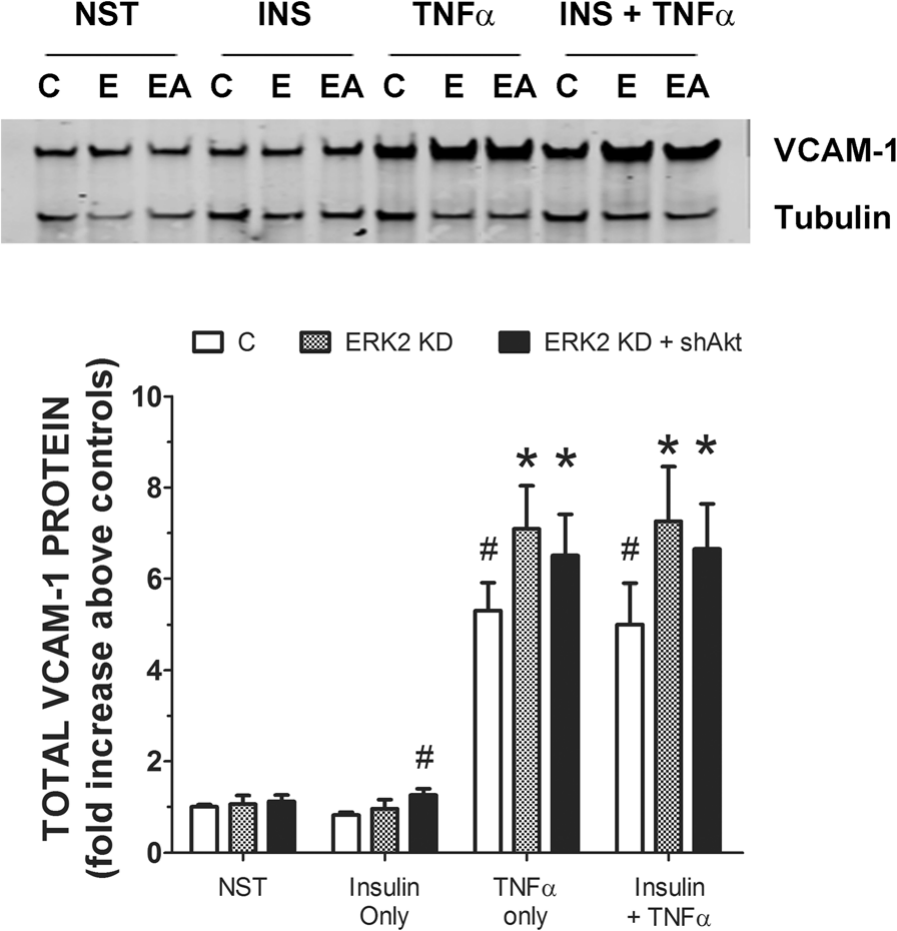
RAEC transfected with shERK2 and shAkt and then stimulated with insulin or TNFα alone or in combination exhibit increased VCAM-1 protein expression greater than stimulated mock transfected cells, but not greater than cells transfected with one shERK2 alone. RAEC were stably transfected with shERK2 and then transiently transfected with shAkt. Subsequently, cells were stimulated with insulin (10 nM, 1 h) or TNFα (10 ng/mL, 6 h) or in combination. (Upper Panel) C, control RAEC, no shRNA. E, cells transfected with shERK2 alone. EA, cells stably transfected with shERK2 and then transiently transfected with shAkt. (Lower Panel) Open bars are control cells not transfected. Stippled bars (ERK2 KD) RAEC stably transfected with shERK2. Black bars (ERK2 KD + shAkt) RAEC stably transfected with shERK2 and transiently transfected with shAkt. “NST”, cells not stimulated with insulin or TNFα. Total VCAM-1 is expressed as fold increase above negative controls and represents the mean ± SEM of five independent experiments. Alpha-tubulin was used to normalize VCAM-1 profiles. #, *P* < 0.05 verses non-stimulated controls. *, *P* < 0.05 versus stimulated controls of similar treatment group without shRNA

We wanted to see if altering the sequence of transfected shRNA would affect the outcome of VCAM-1 expression. In experiments similar to those depicted in Fig. 5, we stably transfected RAEC with shERK2, waited 2 weeks and then transiently transfected the cells with shAkt. Similar to the experiments in which shAkt was stably expressed, ERK2 KD expressing transiently transfected shAkt and stimulated with either insulin or TNFα alone or in combination did exhibit increased VCAM-1 above positive controls (no shRNA) (Fig. 6). However, similar to that observed in Fig. 5, expression of VCAM-1 was not significantly greater in ERK2 KD cells transiently transfected with shAkt as compared to ERK2 KD cells alone.

**Fig. 7:**
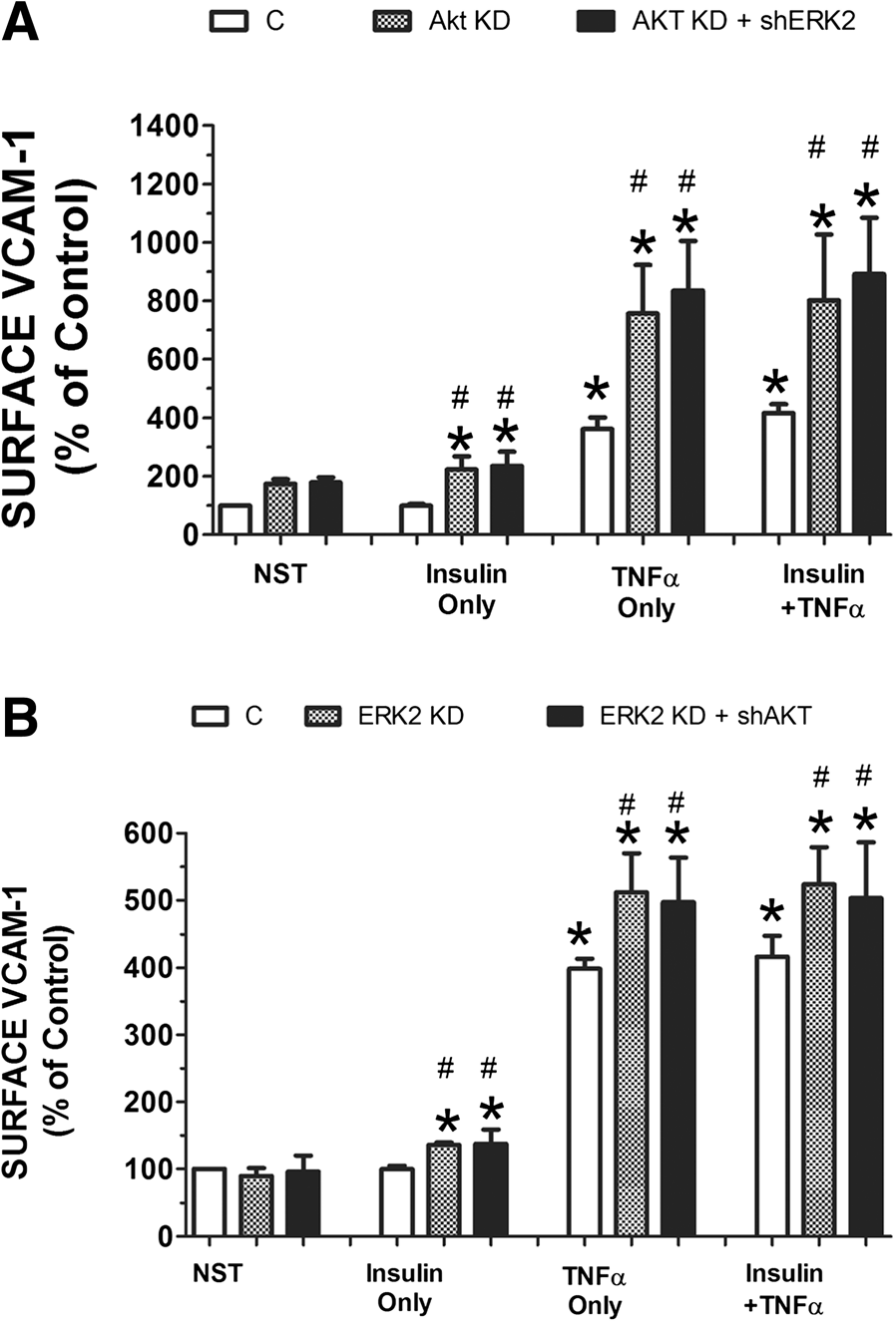
RAEC transfected with shAkt and shERK2 exhibit increased insulin and TNFα-stimulated VCAM-1 at the cell surface as determined by flow cytometery. (Panel **a**) RAEC were stably transfected with shAkt (Akt KD) or (Panel **b**) shERK2 (ERK2 KD) and then transiently transfected with shERK2 or shAkt, respectively. Subsequently, cells were stimulated without or with insulin (10 nM) or TNFα (10 ng/mL) or in combination and surface VCAM-1 was determined by flow cytometry. C, non-transfected RAEC (no shRNA). NST, no stimulation. Akt KD, stable cell lines with shAkt. ERK2 KD, stable cell lines with shERK2. Akt KD + shERK2, stable shAkt cells lines transiently transfected with shERK2. ERK2 KD + shAkt, stable cell lines with shERK2 transiently transfected with shAkt. Surface VCAM-1 is expressed as percent of respective controls (not stimulated, No Stim) and represents the mean ± SEM of five independent experiments. *, *P* < 0.05 compared to their respective unstimulated controls. #, *P* < 0.05 compared to stimulated non-transfected controls

**Fig. 8:**
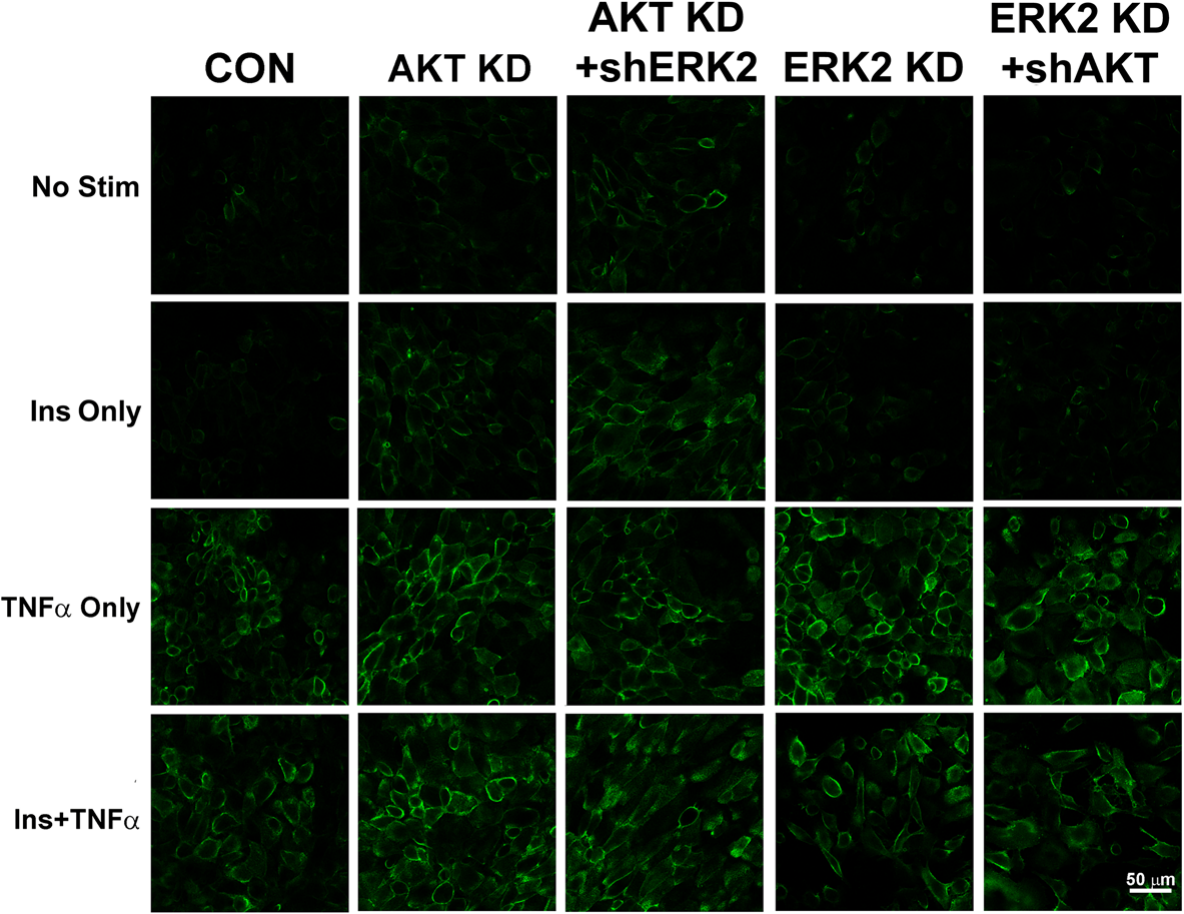
RAEC transfected with shAkt and shERK2 exhibit increased insulin and TNFα-stimulated cell surface VCAM-1 as determined by confocal microscopy. RAEC were plated and stimulated as described in Methods. Surface VCAM-1 was detected by immunocytochemistry as described in Methods and visualized by confocal microscopy. CON, RAEC controls, no shRNA; No Stim, no stimulation by insulin or TNFα; Akt KD, stable cell lines transfected with shAkt. ERK2 KD, stable cell lines transfected with shERK2. shAkt, cells transiently transfected with shAkt. shERK2, cells transiently transfected with shERK2. Scale bar is 50 μm

Finally, we wanted to determine whether increased VCAM-1 at the total cellular protein level equated to increases at the cell surface. We used two different procedures to determine changes in VCAM-1 at the cell surface: flow cytometry and confocal microscopy. Using flow cytometry, both the Akt KD and ERK2 KD cell lines exibited significantly (*P* < 0.05) greater expression of VCAM-1 at the surface in the presence of insulin and/or TNFα than cells mock transfected (Fig. 7). In order to visualize the increases in VCAM-1 at the cell surface, we utilized confocal microscopy and again observed increased VCAM-1 at the cell surface in Akt KD and ERK2 KD cell lines (Fig. 8).

## Discussion

DM is a pervasive disease that affects old and young alike, and is followed by a sequala of effects. Cardiovascular disease and atherosclerosis are major players in the insidious repertoire of DM and their existence is based upon increased amounts of, but not limited to, serum insulin and TNFα. In turn, these biomoleculer signals escalate downstream cellular events such as increased expression of VCAM-1. Equally important are the kinase mediators that transduce the serum and cell surface signals of insulin and TNFα to intracellular events. ERK2 and Akt are members of this large kinase family and although they may not be the only intracellular mediators of these external signals, they appear to be conduits of inflammatory regulation within endothelial cells.

Kinases such as ERK2 and Akt are major players in intracellular signaling [12]. Down regulation of one may have profound effects in intracellular events [13–16]. Loss of positive effectors or negative inhibitors may cause *unknown effects* in the cell and the vasculature. The loss of positive players may cause decreases in positive-regulation of key pathways, thereby decreasing their beneficial effects. Alternatively, the loss of negative effectors (i.e., inhibitors) may cause *the loss of negative-regulation in the same or parallel pathways. In the current report, knock-down of ERK2 and Akt appeared to perturb the physiologic (inhibitory) qualitiy of these kinases, eliciting an upregulation of VCAM-1. Interestingly, decreased expression of both ERK2 and Akt did not have an additive or synergistic effect. This may mean that they are in the same regulatory pathway or other effectors are regulating the expression of VCAM-1 as well.*

The down-regulation of ERK2 and Akt in our study significantly (*P* < 0.05) increased VCAM-1 in cells treated with both insulin and TNFα: a possible scenario in the atherosclerotic vasculature. Kinases are part of common, intracellular pathways that appear to “cross-talk” within the cell [17]. The concert of these signals may keep downstream events in check, whereas their pertubations may cause *unbeknownst* effects.

Inflammation is associated with many diseases such as obesity, cancer, autoimmunity and atherosclerosis [18–22]. The essential cellular events that initiate the inflammatory process in the vasculature are extracellular signals. They in turn *act on* cell surface receptors and instigate intracellular signaling via the kinases. It is these kinases that are instrumental in upregulating the inflammatory markers such as VCAM-1 [23, 24].

Interestingly, other studies have established that pro-inflammatory cytokines such as IL-1β are upregulated by VCAM-1 expression [25]. It will be very interesting to determine if TNFα is upregulated by VCAM-1 at the monocyte as well. If this does occur a positive feed-forward mechanism is possible.

Here we report that not only did insulin and TNFα stimulate *the* expression of VCAM-1, but also that drecreased expression of ERK2 and Akt resulted in increased expression of VCAM-1 at the total protein and cell surface level. Our future studies will evaluate the expression of VCAM-1 in the presence of other inhibitory RNAs; in particular, p38 and JNK. Both p38 and JNK have been shown to be involved with inflammation. Obviously, the knock-down of these two kinases and their effects on VCAM-1 expression will be very interesting and will add to the “cross-talk” picture of the cell. *These current and future studies will benefit strategies that will assist in decreasing the inflammatory effects of atherosclerosis in the vasculature.*

## Conclusions

Insulin and TNFα increased VCAM-1 expression in RAEC. Yet, RAEC transfected with shAkt and shERK2, which cause decreased expression of Akt and ERK2, initiated increases in total and cell surface VCAM-1 protein in the presence of insulin and/or TNFα greater that seen in mock transfected cells.
